# Pericardial calcification in constrictive pericarditis

**DOI:** 10.1186/1865-1380-5-37

**Published:** 2012-10-13

**Authors:** Michel Toledano, Anjali Bhagra

**Affiliations:** 1 Department of Neurology, Mayo Clinic, Rochester, 200 First Street SW, Rochester, MN, 55905, USA; 2 Division of Internal Medicine, Mayo Clinic, 200 First Street SW, Rochester, MN, 55905, USA

## Abstract

**Background:**

A high index of suspicion is required to make the diagnosis of constrictive pericarditis (CP) in patients presenting with cirrhosis and volume overload, as they can otherwise go misdiagnosed for years.

**Methods:**

Case report.

**Findings:**

A 51 year-old man with a history of presumed alcoholic cirrhosis presented to the emergency department with anasarca. Abdominal ultrasound with Doppler demonstrated a nodular cirrhotic liver, but no evidence of portal hypertension or ascites. The chest x-ray, however, was significant for a right-sided pleural effusion and pericardial calcification, suggestive of (CP). Transthoracic echocardiogram and ECG-gated computerized tomography scan of the chest without IV contrast confirmed the diagnosis. The patient was referred to thoracic surgery for definitive pericardiectomy.

**Conclusion:**

The diagnosis of CP is often neglected by admitting physicians, who usually attribute the symptoms to another disease process. Although a multimodality approach is necessary for the diagnosis of CP, this case highlights the utility of chest x-ray, a relatively non-invasive and inexpensive test, in expediting the diagnosis.

## Findings

**Figure 1:**
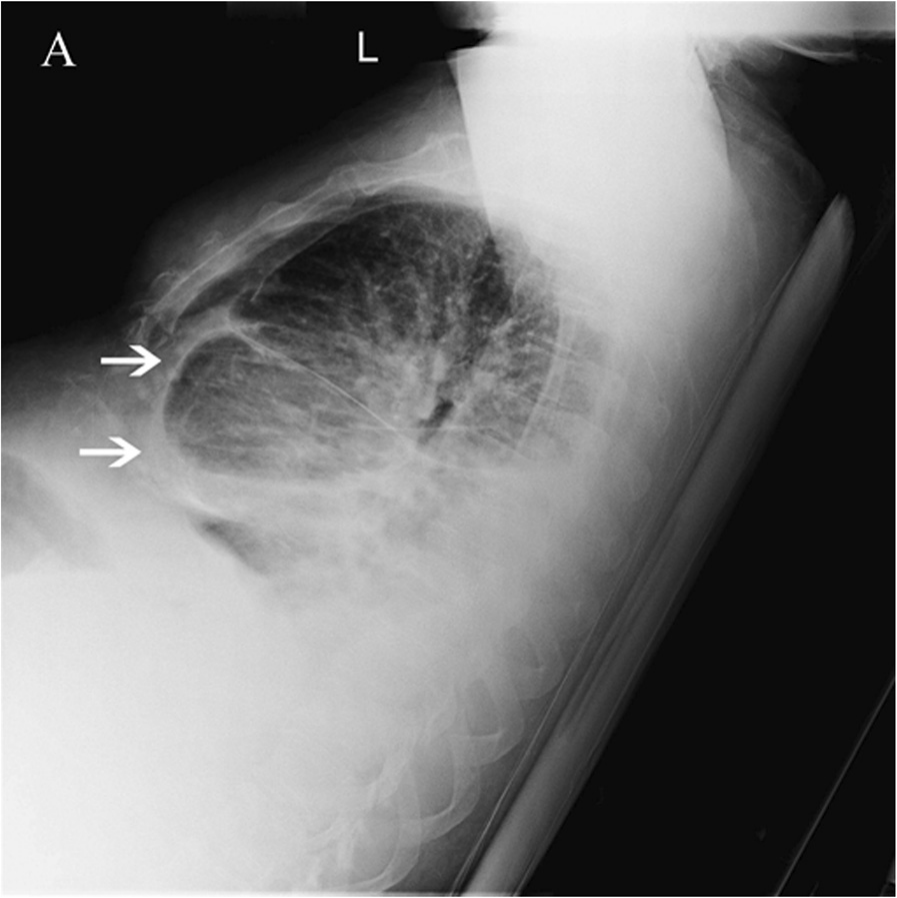
Right-sided pleural effusion and pericardial calcification.

**Figure 2:**
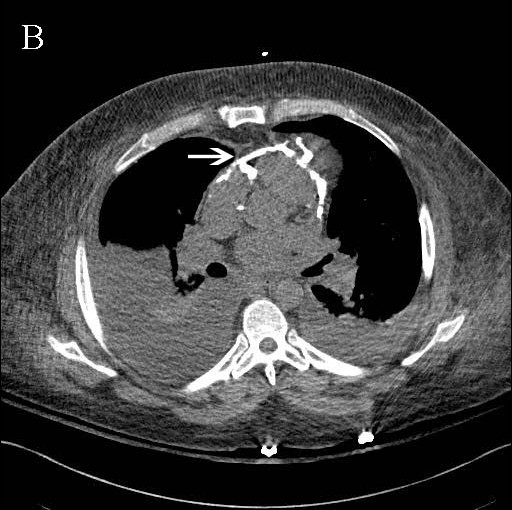
ECG-gated computerized tomography scan of the chest without IV contrast.

A 51-year-old man presented to the emergency department with anasarca. The patient had a history of presumed alcoholic cirrhosis 15 years prior, but denied any alcohol use since that time. Physical examination was remarkable for massive anasarca, with edema prominent up to his chest. Laboratory studies revealed new elevated creatinine and hyponatremia. Liver function tests and urinalysis were unremarkable. Abdominal ultrasound with Doppler demonstrated a nodular cirrhotic liver, but no evidence of portal hypertension or ascites. The chest x-ray was significant for a right-sided pleural effusion and pericardial calcification (Figure 1, arrowheads), suggestive of constrictive pericarditis (CP). A transthoracic echocardiogram was performed and showed severe ventricular septal shift to the left with inspiration, as well as marked diastolic ventricular septal bounce. An ECG-gated computerized tomography scan of the chest without IV contrast confirmed extensive pericardial calcification (Figure 2, arrowhead), diagnostic of CP. The patient was referred to thoracic surgery for definitive pericardiectomy. The diagnosis of CP is often neglected by admitting physicians, who usually attribute the symptoms to another disease process [1]. Although a multimodality approach is necessary for the diagnosis of CP [2], this case highlights the utility of chest x-ray, a relatively non-invasive and inexpensive test, in expediting the diagnosis.

## Competing interests

Both authors declare that they have no competing interests.

## Authors’ contributions

Dr. Toledano made substantive contributions to the design of the study and drafting of the manuscript. Dr. Bhagra made substantive contributions to the drafting and revision of the manuscript. Both authors gave final approval to the version to be published.
